# Standardized cardiovascular magnetic resonance imaging (CMR) protocols: 2020 update

**DOI:** 10.1186/s12968-020-00607-1

**Published:** 2020-02-24

**Authors:** Christopher M. Kramer, Jörg Barkhausen, Chiara Bucciarelli-Ducci, Scott D. Flamm, Raymond J. Kim, Eike Nagel

**Affiliations:** 1 Cardiovascular Medicine, University of Virginia Health System, Lee Street, Box 800158, Charlottesville, VA 22908 USA; 2 Department for Radiology, University Hospital Schleswig-Holstein, Lübeck, Germany; 3 Cardiology, Bristol Royal Infirmary, Bristol, UK; 4 Imaging Institute, and Heart and Vascular Institute, Cleveland Clinic, Cleveland, OH USA; 5 Duke Cardiovascular Magnetic Resonance Center, Duke University Medical Center, Durham, NC USA; 6 Institute for Experimental and Therapeutic Cardiovascular Imaging, University Hospital, Frankfurt, Germany

## Abstract

This document is an update to the 2013 publication of the Society for Cardiovascular Magnetic Resonance (SCMR) Board of Trustees Task Force on Standardized Protocols. Concurrent with this publication, 3 additional task forces will publish documents that should be referred to in conjunction with the present document. The first is a document on the Clinical Indications for CMR, an update of the 2004 document. The second task force will be updating the document on Reporting published by that SCMR Task Force in 2010. The 3rd task force will be updating the 2013 document on Post-Processing. All protocols relative to congenital heart disease are covered in a separate document.

The section on general principles and techniques has been expanded as more of the techniques common to CMR have been standardized. A section on imaging in patients with devices has been added as this is increasingly seen in day-to-day clinical practice. The authors hope that this document continues to standardize and simplify the patient-based approach to clinical CMR. It will be updated at regular intervals as the field of CMR advances.

## Introduction

This document is an update to the 2013 publication of the Society for Cardiovascular Magnetic Resonance (SCMR) Board of Trustees Task Force on Standardized Protocols [1]. Concurrent with this publication, 3 additional task forces will publish documents that should be referred to in conjunction with the present document. The first is a document on the Clinical Indications for CMR [2], an update of the 2004 document. The second task force will be updating the document on Reporting published by that SCMR Task Force in 2010 [3]. The 3rd task force will be updating the 2013 document on Post-Processing [4]. All protocols relative to congenital heart disease are covered in a separate document [5].

The section on general principles and techniques has been expanded as more of the techniques common to cardiovascular magnetic resonance (CMR) have been standardized. A section on imaging in patients with devices has been added as this is increasingly seen in day-to-day clinical practice. The authors hope that this document continues to standardize and simplify the patient-based approach to clinical CMR. It will be updated at regular intervals as the field of CMR advances.

## General principles

### Field strength considerations

Clinical CMR can be performed at different field strengths. 1.5 T systems are currently used for the majority of examinations. An increasing number of studies, however, are being performed at 3 T, with advantages and caveats as noted below.

1. Electrocardiographic (ECG) gating may be more problematic at 3 T than at 1.5 T. In cases where the ECG signal is unreliable, peripheral pulse gating may succeed for acquisitions that are amenable to retrospective gating, such as cine imaging.
2. As a result of improved signal-to-noise ratio (SNR), 3 T may be advantageous for first pass contrast-enhanced perfusion imaging and late gadolinium enhancement. Furthermore, tagging sequences and 4D flow techniques may benefit from imaging at 3 T.
3. Balanced steady-state free precession (bSSFP) is well established as the default method of choice for cine imaging at 1.5 T. At 3 T, however, the increased sensitivity of bSSFP to off- resonance effects tends to worsen dark banding and flow artifacts. To mitigate these artifacts, it may be necessary to perform careful shimming. In rare cases, patient-specific frequency adjustment may be required.
4. Devices that have been tested and determined to be safe at 1.5 T may not be safe at 3 T. Please check specific information relating to CMR safety of devices at higher magnetic field strengths. (Please see section 1.6 below.)

### Equipment considerations

#### CMR scanner

1. MR scanners for clinical CMR should have field strength of ≥1.0 T; however, the typical field strength employed is 1.5 T with a number of centers using 3 T scanners.
2. A cardiac imaging specific surface coil with multiple coil elements (typically ≥8 elements) is highly recommended, and is required to employ parallel imaging techniques that reduce scan and breathhold times.
3. ECG-gating hardware and software are required, and preferably incorporate vector-cardiographic gating. ECG-gating capabilities should include the ability to perform prospective gating, retrospective gating, and triggered gating techniques.

#### Software/pulse sequences

1. Required pulse sequences for CMR examinations: cine bSSFP imaging, rapid multi-slice myocardial perfusion imaging, late-gadolinium enhancement (LGE) imaging, phase-contrast flow quantification imaging, and 3D contrast-enhanced angiographic imaging.
2. Parallel imaging capabilities (e.g., Sensitivity encoding (SENSE), simultaneous acquisition of spatial harmonics (SMASH), generalized autocalibrating partially parallel acquisition (GRAPPA)) are highly recommended to reduce scan and breathhold times.

#### Accessory hardware

1. A CMR-compatible power injector is required for performing rapid myocardial perfusion imaging or contrast-enhanced MR angiographic (MRA) techniques.

### Stress agents

Vasodilator stress perfusion testing is more commonly performed than inotropic stress functional testing.

Vasodilator stress agents:

1. Adenosine:140 μg/kg body weight/min for 2–4 min (consider an increase up to 210 μg/kg body weight/min depending on institutional and local norms if, after 2–3 min, heart rate (HR) does not increase by 10 bpm and or systolic blood pressure does not drop by > 10 mmHg)
2. Dipyridamole: 0.142 μg/kg/min over 4 min
3. Regadenoson: 0.4 mg single injection
4. Adenosine triphosphate (ATP) – 140 μg /kg/min for 3–5 min (consider an increase up to 210 μg/kg body weight/min depending on institutional and local norms if, after 2–3 min, HR does not increase by 10 bpm and or blood pressure does not drop by > 10 mmHg)

Inotropic stress agents:

1. Dobutamine: typical maximum dose 40μg/kg/min ± atropine: 0.25 mg fractions typical (maximal dose 2 mg) (ischemia) or 2.5–10 μg/kg/min dobutamine (viability)

#### Contraindications

Adenosine, dipyridamole, ATP, or regadenoson

- 2nd degree (type 2) or complete atrioventricular (AV) block
- Systolic blood pressure < 90 mmHg
- Severe systemic arterial hypertension (> 220/120 mmHg)
- Sinus bradycardia (heart rate < 40 bpm)
- Active bronchoconstrictive or bronchospastic disease with regular use of inhalers
- Known hypersensitivity to adenosine, dipyridamole, or regadenoson

Dobutamine

- Severe systemic arterial hypertension (≥ 220/120 mmHg)
- Unstable angina pectoris
- Severe aortic valve stenosis (peak aortic valve gradient > 60 mmHg or aortic valve area < 1 cm^2^)
- Complex cardiac arrhythmias including uncontrolled atrial fibrillation
- Hypertrophic obstructive cardiomyopathy
- Myocarditis, endocarditis, or pericarditis
- Uncontrolled heart failure

Atropine

- Narrow-angle glaucoma
- Myasthenia gravis
- Obstructive uropathy
- Obstructive gastrointestinal disorders

#### Patient preparation

1. If applicable for the center, obtain informed consent for the stress test.
2. To fully exert the stress agents’ effects patients should optimally refrain from the following substances/medications for 12-24 hours prior to the examination due to potential of interaction with the stress agent.
  All vasodilating agents: caffeine (coffee, tea, caffeinated beverages or foods - e.g., chocolate, caffeinated medications), theophylline, dipyridamole.
  Dobutamine: ß-blockers and nitrates.
    Note: There is increasing data that the effects of caffeine and nicotine can be overcome by higher doses of adenosine as well as regadenoson.
3. Fasting is not mandatory, but is often advised because recognized adverse effects of stress agents include nausea and vomiting, which may be problematic when lying supine in the restricted space of the scanner.
4. If adenosine is used, it is preferred that two intravenous lines should be available, one for gadolinium based contrast agent (GBCA) and one for adenosine, one in each arm. The preferential site of contrast infusion is antecubital vein, but other veins can be used. The largest cannula should be used for contrast agent. The rate of infusion of contrast agent should be adjusted based on the size of the cannula used.
5. The blood pressure cuff should be used with care taken not to interfere with GBCA or adenosine infusion.
6. For regadenoson, only one intravenous line is required. Many sites reverse the regadenoson with aminophylline 100 mg IV after acquiring stress images. While this may reduce the side effects and return heart rate to baseline immediately, aminophylline also has arrhythmogenic side effects and should thus be used with caution. Side effects usually dissipate after 2–3 min.
7. Side effects are described as less significant with regadenoson than with the other vasodilators; however, the half-life of regadenoson is longer if not actively reversed.

#### Potential adverse effects

Adenosine, ATP, and regadenoson may cause flushing, chest pain, palpitations, and breathlessness. More severe side effects include transient heart block, transient hypotension, or bronchospasm.

Dipyridamole may cause chest pain, headache, and dizziness. More severe side effects are rare and include myocardial infarction, ventricular tachycardia, and transient ischemic attack.

Dobutamine at high doses may cause chest pain and palpitations. More severe complications are rare, including myocardial infarction, ventricular fibrillation, and sustained ventricular tachycardia.

### Stress and safety equipment

1. Monitoring equipment (blood pressure; at least single lead ECG for monitoring of cardiac rhythm; intercom to communicate with patient; for patients with devices - pulse oximetry)
2. Preparation and regular departmental practice for rapid removal of the patient from the scanner
3. Emergency resuscitation policy in place
4. Crash cart with appropriate resuscitative medications, supplies, and equipment with established location outside the scanner room
  - Immediately at hand: ß-blocker (e.g., esmolol or metoprolol), nitroglycerin, aminophylline, bronchodilators, oxygen
  - In an emergency cart: full set of emergency drugs (including drugs such as: epinephrine, ß-blockers, atropine, bronchodilators, antiarrhythmic drugs)
5. For dobutamine – Ability to rapidly review images for assessment of wall motion during image acquisition

### Gadolinium based contrast agent (GBCA) dosing and safety

See Table 1.

**Table 1:** Contrast and chasing bolus doses and injection rates

Indication	Contrast dose (mmol/kg body weight)	Injection rate	Saline chasing bolus	Injection rate
Perfusion	0.05–0.1	3–7 ml/s	30 ml	3–7 ml/s
Late gadolinium enhancement	0.1–0.2		20 ml	
Angiography (carotids, renals, aorta)	0.1–0.2	2–3 ml/s	20 ml	2–3 ml/s
Time-resolved angiography	0.05	3–5 ml/s	30 ml	3–5 ml/s
Peripheral angiography	0.2	first 10 ml @ 1.5 ml/s, rest @ 0.4–0.8 ml/s	20 ml	0.4–0.8 ml/s

Notes:

1. Volumes and injection rates vary depending on the contrast agent and scan protocol.
2. Injection rates are different for 1 mmol/ml contrast agents (e.g., gadobutrol) and 0.5 mmol/ml agents. As a guideline, divide the given injection rates by a factor of 2 for the 1 mmol/ml formulation.
3. GBCA contrast agents with higher relaxivity require smaller doses.

Safety considerations:

1. More than 300 million GBCA doses have been applied worldwide since 1988 [5]. GBCAs provide crucial medical information in many applications and have an excellent safety profile.
2. However, nephrogenic systemic sclerosis (NSF) and long-term gadolinium retention in the brain have resulted in regulatory actions.
3. In 2017 the European Medicines Agency (EMA) decided to suspend the marketing authorizations of all multipurpose linear GBCAs and to continue to use all macrocyclic GBCAs. The United States Food and Drug Administration (FDA) maintained all GBCAs but decided that warnings have to be included in the prescribing information that communicates the greater risk of gadolinium deposition when using linear GBCAs. Additionally, product information updates should incorporate risk mitigation steps and a Medication Guide for each product.
4. Healthcare professionals should consider the retention characteristics of each agent when choosing a GBCA, and particularly for patients who may be at higher risk for gadolinium retention or NSF [6].
5. The dose of GBCA in all CMR applications should be as low as possible to achieve adequate image quality, and the prescribing information of the products as well as the institutional, regional or national guidelines have to be respected.
6. Noncontrast techniques should be considered as alternatives for contrast-enhanced techniques whenever possible.

### Imaging patients with cardiac devices (pacemakers and defibrillators)

1. Safety
  - Follow manufacturer and institutional guidelines for patients with MR-conditional devices and non-conditional devices.
  - Patients with cardiac devices implanted < 6 weeks before the CMR scan should in general not be scanned, unless the clinical indication is compelling and informed patient consent is obtained.
  - Patients with abandoned or epicardial leads should in general not undergo scanning, unless the clinical indication is compelling and informed patient consent is obtained.
  - Device programming will depend upon pacer dependence and recommendations by electrophysiology specialists. In general, if the patient is pacemaker dependent, the pacemaker should be programmed to asynchronous mode and if not pacer dependent, it should be programmed to nonpacing or inhibited mode.
  - Devices should undergo interrogation before and after the CMR scan.
  - Trained personnel should be available for patient, ECG, and oxygen saturation monitoring during the scan.
  - Resuscitative equipment should be available close to the scanner room.
2. Imaging
  - Placing the arm associated with the side of the pacemaker generator over the head during the scan may improve image quality.
  - Imaging during deep inspiration may improve image quality.
  - If significant artifact is present on bSSFP cine imaging, gradient echo cine imaging may be preferred.
  - To reduce device-related image artifact, wideband late gadolinium enhancement imaging may be useful, particularly in the presence of an implanted cardiodefibrillator (ICD).

## General techniques

### Left ventricular (LV) structure and function

**Fig. 1:**
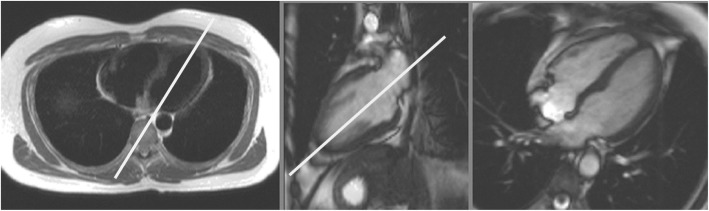
Left – Black blood axial scout image through the base of the left ventricle (LV) and right ventricle (RV). Planning of the 2 chamber long-axis is shown by the white line. Center – White blood 2 chamber long axis scout image. Planning of the 4 chamber long-axis is shown by the white line. Right **–** White blood 4-chamber long axis scout image

**Fig. 2:**
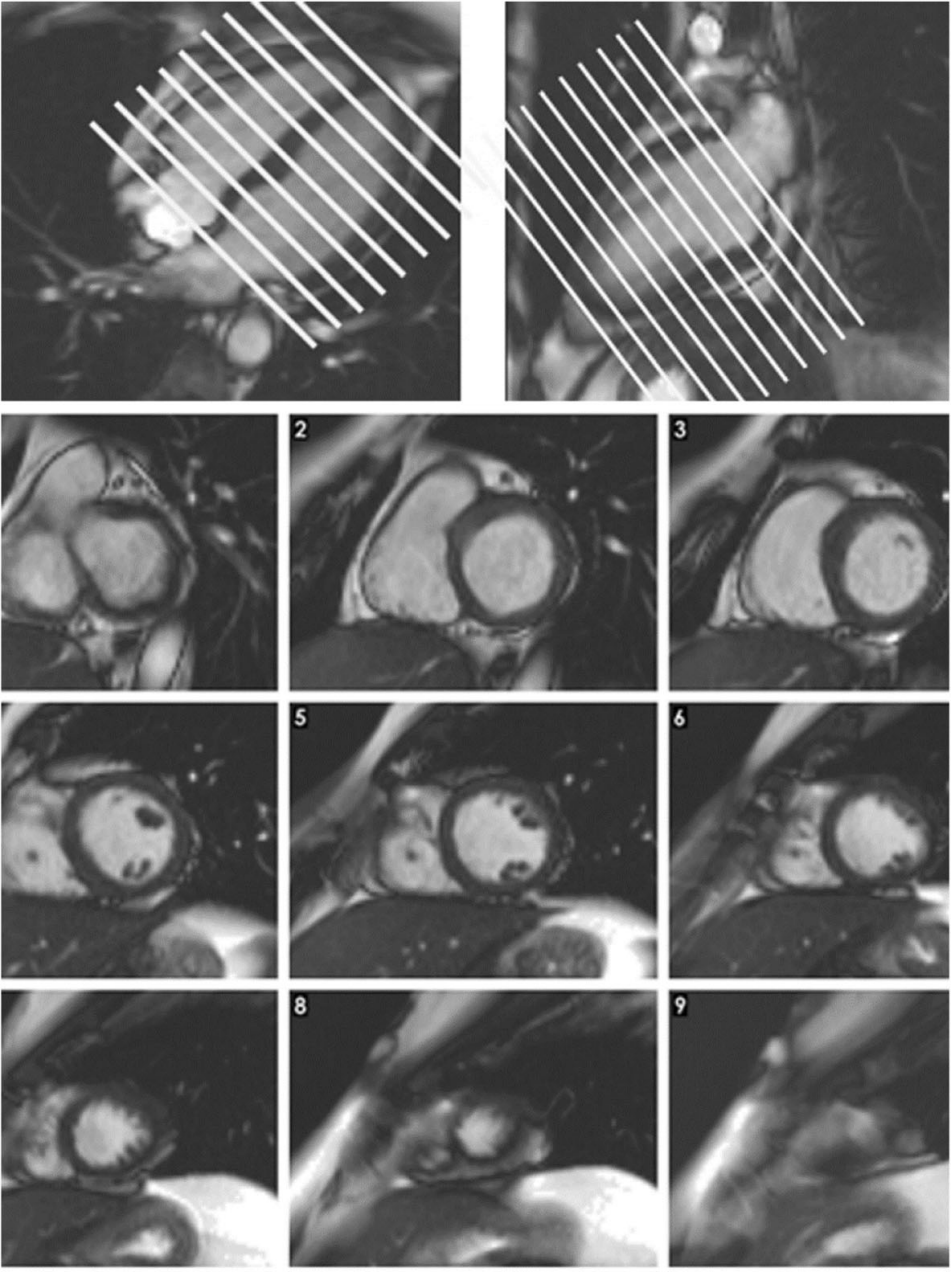
Top – Planning of the short axis image plane parallel to the mitral valve in the 4 chamber long axis plane (left) and 2 chamber long-axis plane (right). Bottom panel – 9 short axis cine slices shown from base (top left) to apex (bottom right)

**Fig. 3:**
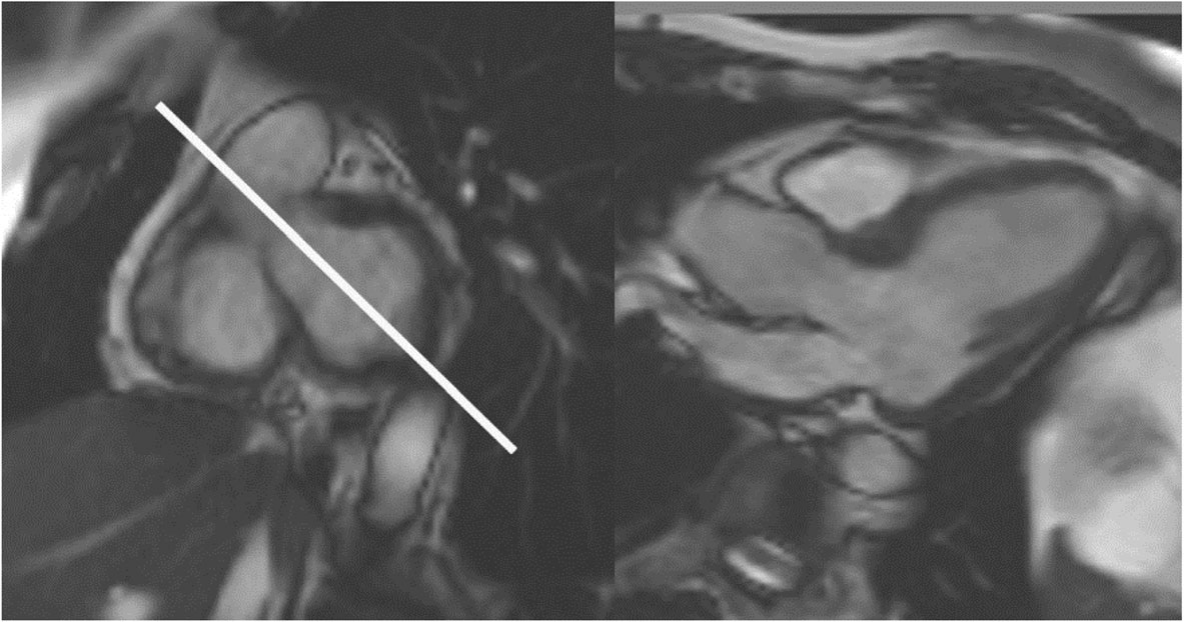
Left – Basal short axis cine image. Planning of the 3-chamber long axis is shown by the white line. Right – 3-chamber long axis cine image

1. Scout imaging – transaxial, coronal, sagittal – these are in general single heartbeat acquisitions acquired in 1 breathold.
2. Transaxial (8–10 mm) set of bSSFP or fast spin echo (FSE) images through the chest. These are single-shot, single heartbeat images with a set acquired in 1–2 breathholds.
3. Scout to line up short axis images – cine acquisitions are preferable to single shot as long axis motion and inflow should be visualized
  - LV two chamber (vertical) long axis prescribed orthogonal to transaxial scouts aligned through the apex and center of the mitral valve (Fig. 1)
  - Four chamber (horizontal) long axis aligned orthogonal to the 2 chamber long axis, passing through the center of the mitral valve and left atrium and continuing through the long axis of the LV. (Fig. 1)
4. bSSFP is the method of choice for cine imaging as it provides high SNR and excellent contrast between myocardium and blood pool
  - At 3 T, SSFP cine images may be compromised by artifact and spoiled gradient-echo sequences can be considered as an alternative
  - Strategies to reduce or move banding artifact include shimming, reducing the TR, and adjusting the RF frequency (frequency ‘scout’ sequence can be helpful for this)
  - Cine images are acquired during a breath-hold. Breath-hold on expiration provides more consistent positioning but inspiratory breath-hold may be more comfortable and easier to sustain for some patients.
5. bSSFP short axis cine images (Fig. 2)
  - Acquired from the base of the LV through the apex.
  - The first short-axis cine plane should be planned using the 4- and 2-chamber long-axis views, and it should be perpendicular to the long-axis of the body of the LV. This plane might not always be parallel to the mitral valve plane.
  - Slice thickness 6–8 mm, with or without 2–4 mm interslice gaps (to make a total of 10 mm).
  - Temporal resolution ≤45 ms between phases to optimize evaluation of wall motion
  - Parallel imaging or compressed sensing used as available to shorten scan time.
6. bSSFP long axis cine images
  - The 4-chamber long-axis view is prescribed from the 2-chamber long-axis view through the apex and center of the mitral and tricuspid valves. This can be modified and/or cross-checked on basal short-axis views, to have the plane cross the acute margin of the right ventricular (RV) free wall and perpendicular to the interventricular septum.
  - The 2-chamber LV view is prescribed from the vertical long-axis scout already acquired with modification to pass through the anterior and inferior myocardial walls.
  - The 3-chamber LV view is prescribed passing through the apex, the center of the mitral valve and aligned with the center of LV outflow tract (LVOT) to aortic valve, as seen on a basal short axis cine. (Fig. 3)
  - Optional – more than 3 long axis views can be obtained.
7. Real-time cine imaging (optional)
  - To assess ventricular interdependence or for patients with irregular rhythms or inability to breathhold that preclude standard gated cine imaging, real-time cine imaging (using a variety of different k-space acquisition approaches) may be used to assess LV function.
  - Temporal resolution ≤60 ms between phases is preferable if available.
  - Absolute LV volume quantification is not always possible using real-time cine imaging as quantitation is typically less accurate and precise.

### Right ventricular (RV) structure and function

**Fig. 4:**
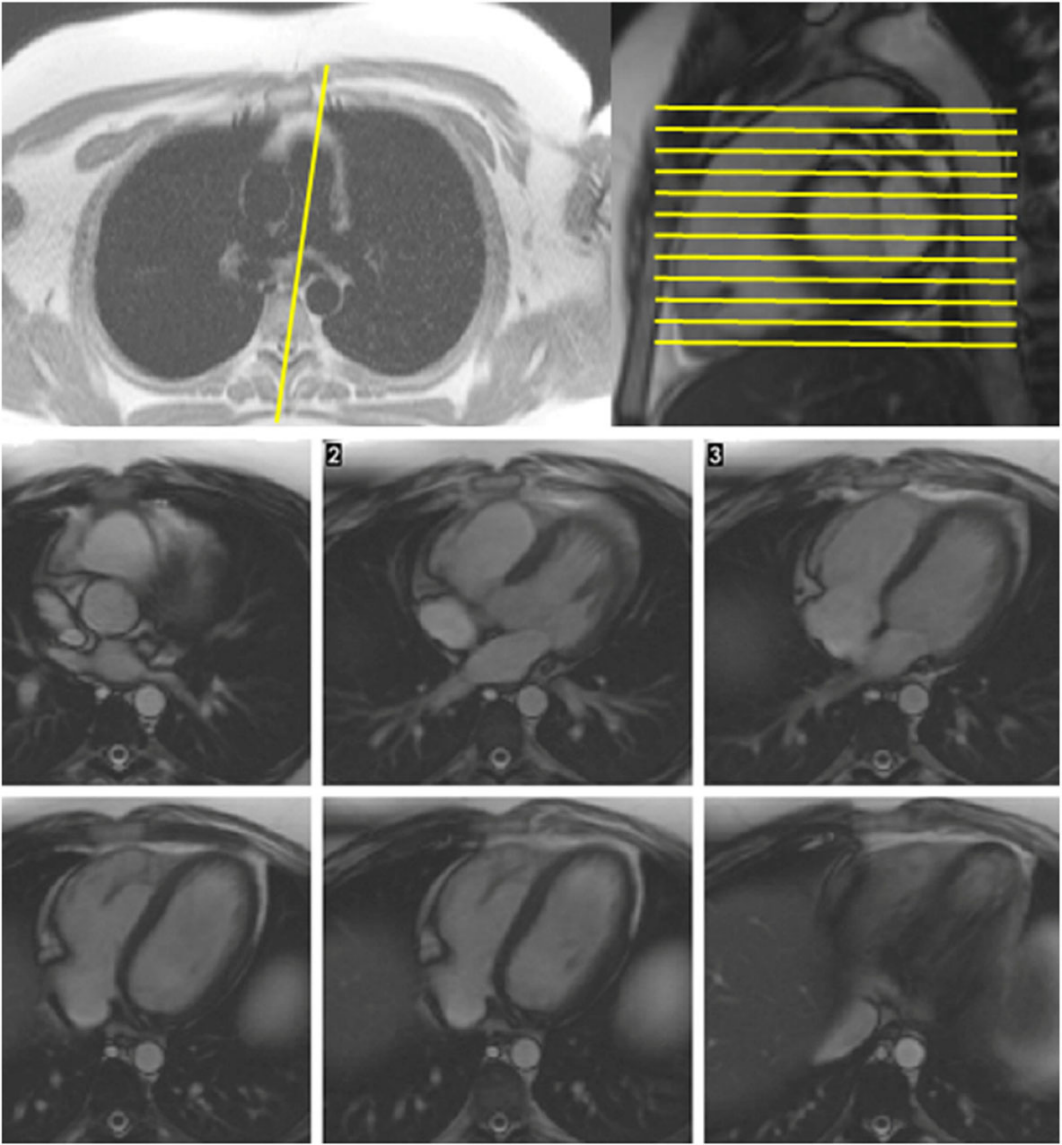
Top left – Axial black blood scout image through the pulmonary artery. Planning of the RV outflow tract (RVOT) view is shown by the yellow line. Top right – RVOT cine image. Planning of axial stack of images is shown by the yellow lines. Bottom panel – 6 sequential axial images are shown from the RVOT (top left) to the inferior pole of the RV (bottom right)

1. RV short-axis views can be obtained in a similar fashion to LV structure and function. If the short-axis is used for quantification, it is important to place the basal short axis slice immediately on the myocardial side of the RV.
2. Long-axis images should include an RV vertical long-axis view aligned with tricuspid valve inflow and an RV outflow tract view (sagittal or oblique sagittal plane through the pulmonary valve). (Fig. 4)
3. Transaxial stack of cines covering the RV can be considered as an alternative for RV volumetry. (Fig. 4)

### First pass perfusion

**Fig. 5:**
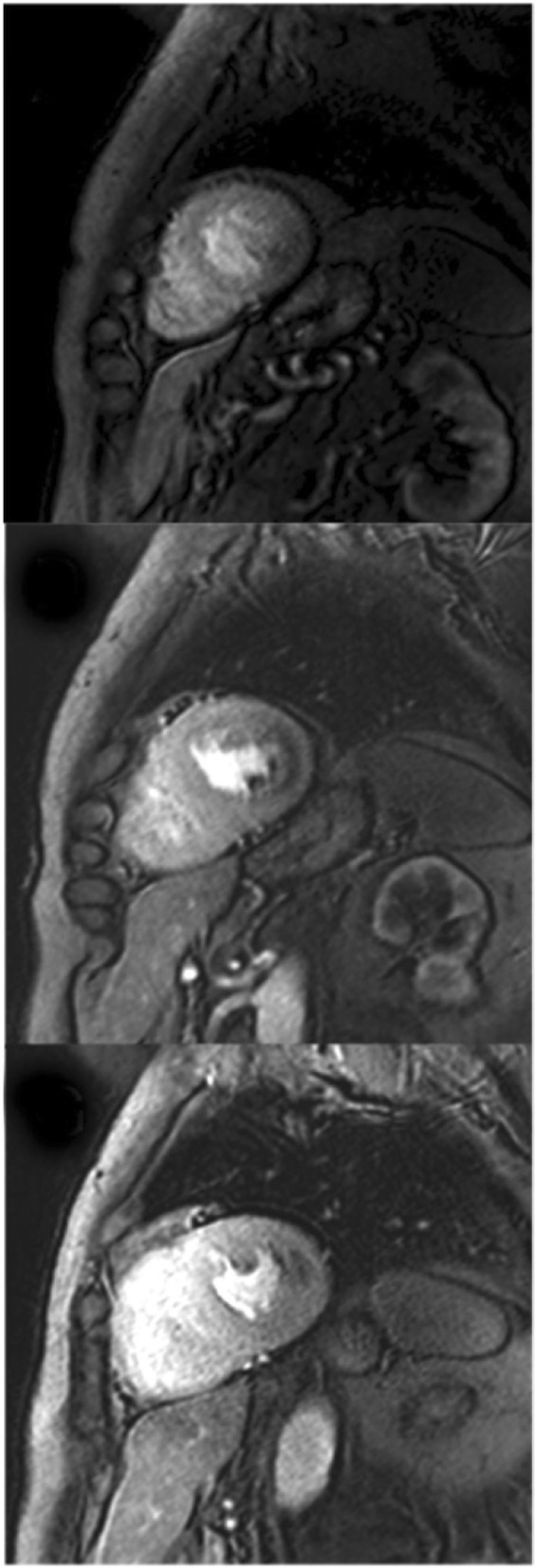
Three short axis images (apex at top, mid in the middle, and base at the bottom) acquired during the first pass of gadolinium based contrast agent (GBCA) through the myocardium. Note the perfusion defect in the lateral wall in the mid and basal slices

1. Scout imaging as per LV structure and function
2. Pulse sequences: Typically saturation-recovery imaging with bSSFP, gradient echo (GRE), or GRE-echo planar (GRE-EPI) hybrid readout
3. Short-axis view imaging (at least 3 slices per heart beat) (Fig. 5)
  - For ischemia evaluation, should obtain data every heart beat, if possible.
  - Slice thickness 8–10 mm
  - Parallel imaging, if available
  - In-plane resolution, ~ < 3 mm
  - Readout temporal resolution ~ 100–125 ms or shorter as available
  - Contrast is given (0.05–0.1 mmol/kg, 3–7 ml/s) followed by at least 30 ml saline flush (3–7 ml/sec)
  - Breathhold starts before contrast reaches the LV cavity.
  - Acquire sufficient number of images to ensure contrast has passed through the LV myocardium (typically at least 50–60 heart beats, but patients with low cardiac output may require more images to be acquired)
  - Optional - Images may also be obtained free breathing, particularly if motion correction sequences are available.

### Late gadolinium enhancement (LGE)

**Fig. 6:**
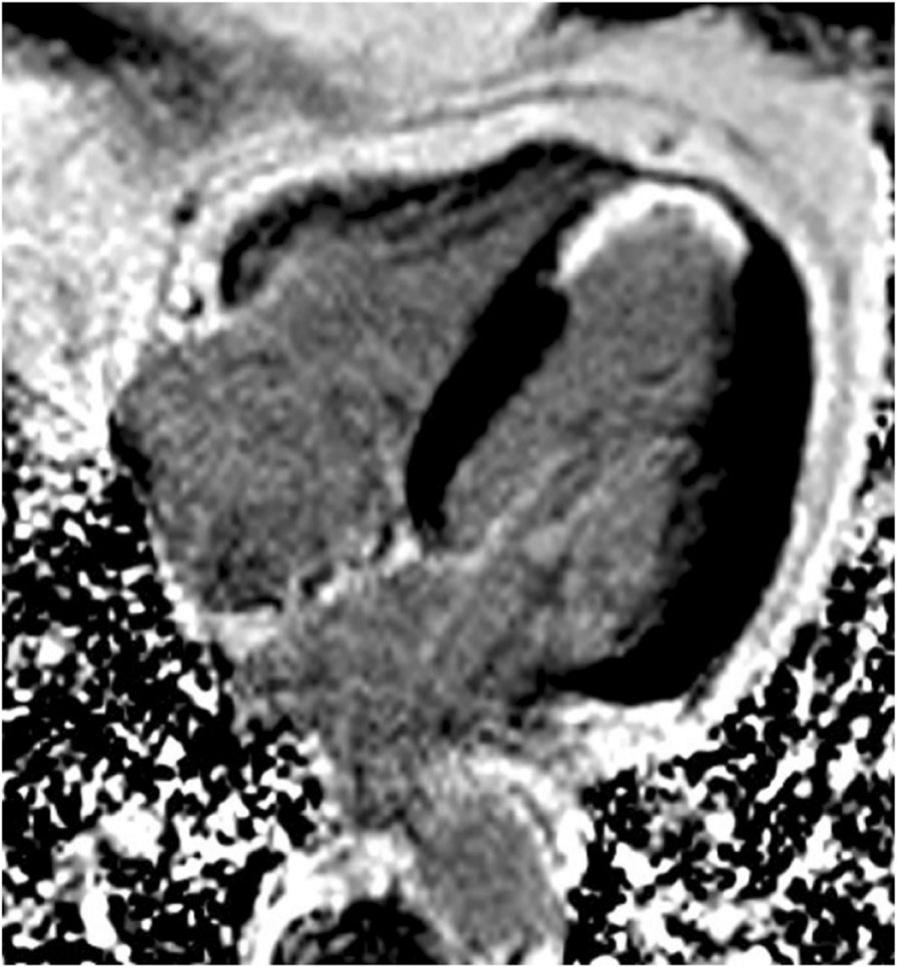
Four-chamber long axis inversion recovery gradient echo late gadolinium enhanced image from a patient with a 50–75% transmural apical septal and apical myocardial infarction

1. Pulse sequences:
  - 2D segmented inversion recovery GRE or bSSFP, Phase-Sensitive Inversion-Recovery (PSIR), or 3D sequences are preferred in appropriate patients with satisfactory breathholding ability and if SNR is sufficient.
  - Single-shot imaging (bSSFP readout) performed as an optional second set or as backup for patients with irregular heart beat, and/or difficulty breath holding.
2. Need at least 10 min wait after GBCA injection (for dosing see Table 1). Note - the delay may be < 10 min if lower gadolinium doses are used as blood pool signal falls below that of late enhanced myocardium. Images are generally acquired during diastolic standstill. Also note – if stress and rest perfusion imaging is performed, the wait should only be approximately 5 min after the 2nd injection of contrast.
3. Same views as for cine imaging (short- and long-axis views) (Fig. 6)
4. Slice thickness, same as for cine imaging
5. In-plane resolution, ~ 1.4–1.8 mm
6. Acquisition duration per R-R interval below 200 ms, but should be less in the setting of tachycardia to avoid image blurring.
7. Inversion time (TI) set to null normal myocardium. A “TI scout”, which is a cine sequence with an inversion pulse that is played at the beginning of the R-wave, can be used as a rough guide to set the TI. However, the TI scout sequence usually does not have the identical readout parameters as the segmented LGE sequence and hence the correct TI may be up to 50 ms different between the two sequences. Alternatively, a PSIR sequence can be used, which obviates the need for a precise setting of the TI.
  - Imaging using a “long-inversion” time (~ 550 ms at 1.5 T and 850 ms at 3 T) can be helpful in distinguishing no-reflow zones or mural thrombus from viable myocardium.
  - Imaging using a short inversion time (~ 200 ms) and PSIR can be helpful in distinguishing subendocardial scar.
8. Read-out is usually every other heartbeat, but should be modified to every heartbeat in the setting of bradycardia (< 60 bpm), and every third heartbeat in the setting of tachycardia (> 100 bpm) or arrhythmia.
9. Dark-blood LGE imaging (optional)
  - If available, flow independent “dark-blood” techniques may be helpful in differentiating subendocardial LGE from blood-pool compared with conventional LGE imaging.
  - Settings, except for inversion time (which is set according to the specific sequence that is used), are similar to conventional LGE imaging.

### Stress perfusion (vasodilator)

1. LV structure and function (alternatively this can be performed between stress and rest perfusion, although performance immediately after GBCA infusion may reduce the contrast of the blood-endocardium interface)
2. Adenosine stress perfusion imaging. Option – initial adenosine infusion may be performed with the patient outside the bore of the scanner, and move the patient inside for the second half of the infusion.
  - First pass perfusion
  - During last minute of adenosine, GBCA is injected
  - Use same approach for dipyridamole
3. Alternatively: Regadenoson stress perfusion imaging (bolus injection of 0.4 mg).
  - First pass perfusion
  - Approximately 45–60 s after regadenoson injection, inject GBCA
4. Rest Perfusion
  - Need at least 10 min wait for to wash out from stress perfusion imaging. During this period cine imaging can be completed (e.g., long-axis views).
  - Perfusion imaging repeated without adenosine/regadenoson using same dose of GBCA
  - Depending on institutional policy and experience, rest perfusion can be omitted. There is increasing data that rest scanning adds little information and should be omitted whenever possible.
  - Additional GBCA may be given as needed for late gadolinium enhancement (for a total of 0.1–0.2 mmol/kg)
5. Late Gadolinium Enhancement
  - Need to wait at least 5 min after rest perfusion if performed
6. Optional - Quantitative perfusion imaging
  - Consider using a dual bolus or dual sequence approach to reduce the effect of nonlinearity between contrast agent concentration and signal intensity.
  - Consider adding proton density images before the contrast injection. This can be used as baseline correction for full quantification but requires specific scanner software that may not be available on all scanners.

### Stress function (dobutamine or exercise)

1. LV structure and function
2. Dobutamine stimulation (See 1.2, Stress agents)
  - Increase the dobutamine in increments of 10 μg/kg body weight/minute every 3 min starting at 10 μg/kg body weight/minute until the target heart rate [85% x (220-age)] is reached.
  - Add atropine in 0.5 mg incremental doses if the heart rate response is inadequate.
  - Repeat 3 short-axis and 3 long-axis cine views during each increment. These can be obtained with breathhold or real-time at lower heart rates, but at higher heart rates, breathhold acquisitions are recommended due to the ability to improve the temporal resolution.
  - Continuous ECG monitoring and blood pressure measured during each stage.
  - View cine loops immediately after they are acquired.
  - Adapt the bSSFP cine sequence to optimize temporal resolution as needed as the heart rate increases.
  - Stop the test for a new wall motion abnormality, a serious side effect, or achievement of the target heart rate.
3. Alternative – treadmill exercise with a CMR-compatible treadmill in the scanner room with imaging (3 short-axis and 3 long-axis cine views) performed at baseline and after peak exercise. The temporal resolution of the bSSFP cine sequence will need to be shortened for the post-exercise scans.
4. Alternative – supine bicycle exercise with a CMR-compatible ergometer in the scanner room/scan table with imaging (3 short-axis and 3 long-axis cine views) performed at baseline and after peak exercise. The temporal resolution of the bSSFP cine sequence will need to be shortened for the post-exercise scans.

### Blood flow quantitation

**Fig. 7:**
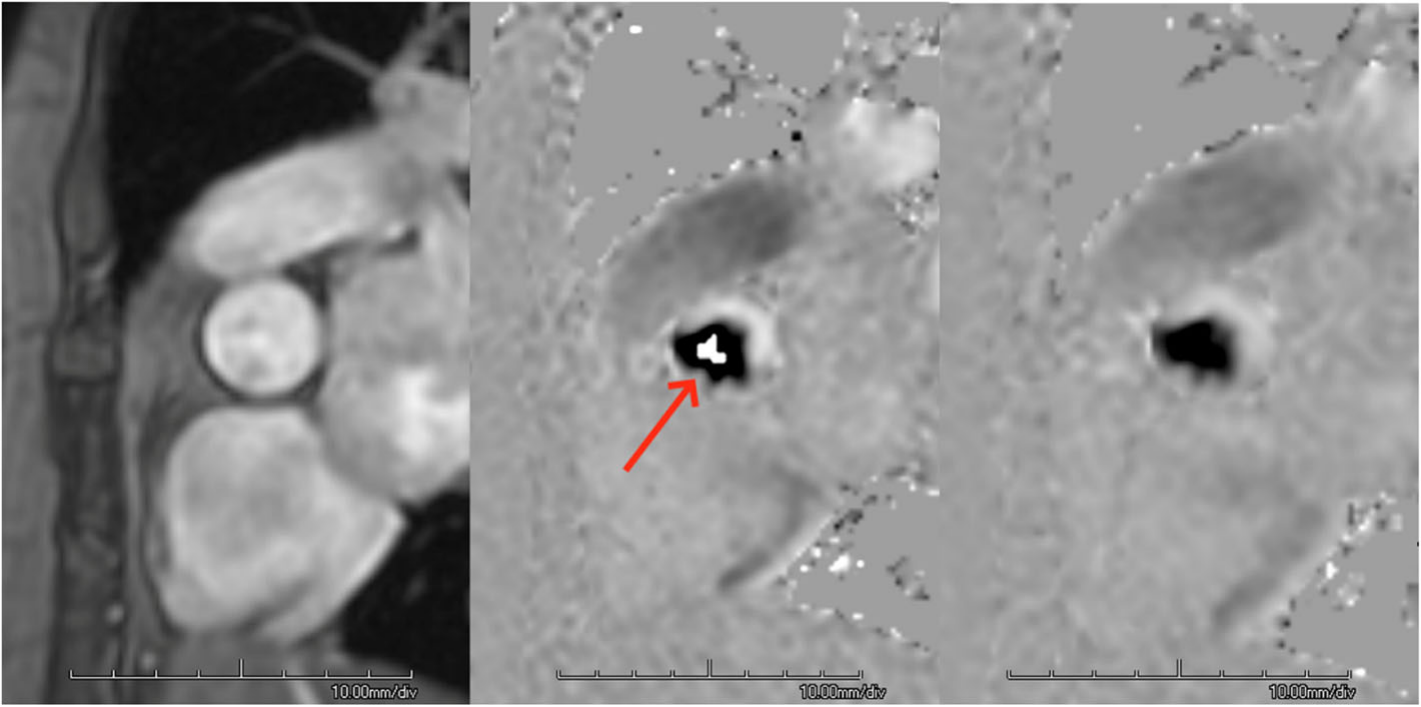
Velocity-encoded flow quantitation sequence acquired at the sinotubular junction in a patient with aortic stenosis. The initial sequence (Magnitude [left] and velocity [center] images) was acquired with a VENC of 250 cm/sec, which was too low, as aliasing (red arrow) is evident. The sequence was reacquired (right) with a VENC upward adjusted to 350 cm/sec, and aliasing is no longer present

1. Usually performed as part of other cardiovascular protocols. Available scout images can be used. Best if vessel of interest is depicted in two orientations or MRA can be reformatted on the scanner for further planning (e.g., additional bSSFP, contrast enhanced (CE)-MRA, or single-shot black blood scouts are helpful)
2. Sequence: one-direction (“through-plane”) motion-encoded cine gradient echo sequences are typically applied (Fig. 7)
3. For optimal results, the imaging plane should be
  - centered in the vessel of interest
  - aligned orthogonally to the expected main blood flow direction in two spatial directions
  - centered in the iso-center of the scanner
4. Imaging parameters: slice thickness 5-8 mm; in-plane resolution at least 1/10th of the vessel diameter. Velocity encoding sensitivity (V_enc_) has to be adapted to the expected velocities – the lowest available velocity without aliasing should be used. After each scan, phase difference images have to be checked for aliasing. If aliasing is present, V_enc_ settings need to be increased accordingly. If available, a velocity scout may allow optimal setting of the V_enc_.
5. A temporal resolution of ≤50 ms between phases is preferable. The temporal resolution should be calculated as the time between frames that are actually acquired. Many vendors allow the creation of extra frames by image interpolation, which only improves the temporal resolution artificially. Retrospective gating covers the entire cardiac cycle and is more convenient, but may obscure inaccuracies related to arrhythmia.
6. For read-out, k-space segmentation over multiple heart beats is used to limit the acquisition time to a breath hold period. Alternatively, navigator-based non-breathhold techniques can be applied to improve the temporal or spatial resolution if necessary. Also, free-breathing approaches with multiple signal averages (NEX, NSA) have been proven useful in patients with limited breathholding capabilities.
7. The echo time (TE) should be set to shortest, particularly when stenoses are imaged.
8. If available, consider a 4D Flow CMR acquisition. 4D Flow CMR is becoming more readily available, it has been shown to provide unique insight in and select clinical settings.

### Advanced tissue characterization

The area of tissue characterization is a rapidly developing field and the pulse sequences available on different vendor platforms vary significantly. The acquisitions are similar between 1.5 T and 3 T, although measured values are often different and may also be site specific. Thus, listed below are general guidelines only as standardization continues to advance. Normal values should be developed at individual institutions. Manual shimming prior to imaging should be considered for optimal performance, particularly at 3 T. For detailed recommendations, please see [7].

1. T1 mapping
  - Native T1 mapping is performed in the absence of contrast agents.
  - Look Locker imaging (modified Look Locker Inversion recovery (MOLLI) or shortened MOLLI (ShMOLLI) or equivalent) should be used.
  - Diastolic acquisition is best with the exception of atrial fibrillation in which systolic acquisition may be preferred. In patients with higher heart rates, specific sequences designed for these heart rates should be used.
  - Source images should be checked for motion/artifact and imaging repeated if this occurs.
  - Slice thickness: 6–8 mm, in-plane resolution ~ 1.6–2.0 mm
  - The number and orientation of slices obtained will depend upon the indication. At least one short-axis map should always be obtained.
  - For extracellular volume measurements, T1 mapping should be performed prior to contrast and at least 1 time point between 10 and 30 min post contrast bolus
  - The hematocrit should be measured, ideally within 24 h of imaging, for the most accurate extracellular volume fraction (ECV) measurement.
2. T2 mapping and T2-weighted (T2w) imaging
  - Mapping - if quantitation is desired and sequence is available
    i. Multiple alternatives exist, such as T2-prepared single-shot bSSFP sequence acquired with different T2 prep time, gradient and spin echo (GraSE) or FSE-based pulse sequences.
    ii. Motion correction as needed
    iii. Slice thickness: 6-8 mm, in plane-resolution ~ 1.6–2.0 mm
    iv. The number and orientation of slices obtained will depend upon the indication. Short-axis maps should always be obtained.
  - T2w Imaging
    i. Black blood T2w short Tau inversion recovery (STIR)
      1. Potential pitfalls – bright signal in areas of low flow, signal dropout due to motion, and low signal in regions with intramyocardial hemorrhage.
    ii. Bright blood T2w sequences
      1. T2-prepared single-shot bSSFP sequence
      2. FSE-bSSFP hybrid is an alternative
      3. Potential pitfall – bright signal may obscure endocardial border
3. T2* mapping
  - T2* images should be obtained prior to contrast administration.
  - The pulse sequence is a single breathhold, gradient-echo, multi-echo scan with a series of 6–9 echo times beginning at ~ 2 msec and extending to ~ 18 msec, with each echo iteratively spaced by ~ 2 msec. A delay time of 0 msec after the R wave typically is used.
  - Optional - In patients with severe iron deposition a pulse sequence with shorter echo spacing could be helpful to accurately determine T2* values: a series of 6–9 echo times beginning at ~ 1 msec and extending to ~ 12 msec, with each echo iteratively spaced by ~ 1 msec.
  - A single mid-ventricular short-axis image is acquired.
  - Slice thickness of 8–10 mm; in-plane resolution, ~ 1.6–3.0 mm
  - (Optional) An imaging sequence similar to the above, though non-ECG-gated, is acquired in the axial orientation through the mid portion of the liver to evaluate hepatic iron deposition. The absence of ECG-gating will allow for closer spacing of iteratively advanced echo times, and therefore a greater number of echoes will be acquired.

### Rapid protocols

Rapid protocols have been developed for 1.5 T scanners and have been successfully applied for cardiomyopathy evaluations. In addition to cardiomyopathies, additional indications for which rapid protocols could be applied include chronic ischemic heart disease to assess viability and hypertensive heart disease, This protocol minimizes exam times and maximizes cost-effectiveness. The protocol as tested is as follows:

1. Localizers, 2 chamber scout image, 3 slice short axis stack scout images, and a transverse dark blood single shot FSE stack for anatomic evaluation.
2. Cine imaging: four, two, three chamber and aortic valve segmented k-space cine acquisitions.
3. Contrast injection of appropriate dose of GBCA
4. Cine imaging: short-axis cine stack (7-mm slice thickness, 3 mm interslice gap) segmented k-space cine acquisitions.
5. LGE imaging:
  - Optional sequence to determine optimal inversion time
  - Segmented k-space LGE acquisitions in standard long axis and short axis planes with phase sensitive and magnitude reconstructions.

## Disease specific protocols –

### Ischemic heart disease

CMR can be uniquely helpful in differentiating between ischemic and various nonischemic forms of acute myocardial injury. Even after the diagnosis of myocardial infarction (MI) has been made, CMR may be helpful in identifying residual viability, stunning, and microvascular damage. In addition, post-MI sequelae, including LV thrombus, LV aneurysm or pseudoaneurysm formation, and pericarditis are easily identified.

#### Acute MI or acute coronary syndromes

1. LV structure and function
2. Advanced tissue characterization - optional, although frequently used to assess edema/inflammation that can accompany acute necrotic injury
3. Optional - First pass perfusion (only at rest). Consider stress if culprit vessel has already been revascularized to evaluate for ischemia in the non-infarct territories
4. Optional - Early gadolinium enhancement, i.e. within the first 1–3 min after contrast infusion to look for early microvascular obstruction (MVO)
5. LGE

#### Chronic ischemic heart disease and viability

General purpose of CMR is to document baseline LV morphology, contractility, viability, and (often) ischemia. Follow-up imaging can be helpful in assessing changes in ventricular remodeling as well as scar and/or ischemia burden following clinical events and/or medical therapeutic interventions. Detection of LV thrombi is also important.

1. LV structure and function
2. Advanced tissue characterization - optional, although may be used to exclude other potential pathologies
3. Optional - low dose dobutamine with 5–10 min infusion of 2.5–10 μg/kg/min of dobutamine to assess contractile reserve identified as improvement in wall thickening
4. Optional - vasodilator stress-rest perfusion or high dose dobutamine functional imaging to determine the presence of inducible ischemia
5. LGE

### Nonischemic heart disease

#### Hypertrophic cardiomyopathy (HCM)

Goals of imaging HCM include measuring LV mass and volumes, global function, and maximal wall thickness (by cine imaging), assessing scar (LGE and T1 mapping) and measuring the LVOT gradient if present.

1. LV structure and function
2. LVOT flow imaging using bSSFP cine imaging in a 3-chamber view examining for turbulence and systolic anterior motion of the mitral valve or chordae, and phase velocity measurements for gradient (using either in-plane phase-velocity imaging in the 3-chamber view, or through plane phase-velocity measurements perpendicular to the LVOT) if LVOT obstruction is present
3. Advanced tissue characterization - optional, although frequently used
4. Optional – consider vasodilator stress perfusion if underlying ischemia is being considered
5. LGE

#### Hypertensive heart disease

Goals of imaging hypertensive heart disease include assessing LV mass, wall thickness, volumes, global function (by cine imaging), and scar (LGE and T1 mapping).LV structure and function

1. Advanced tissue characterization - optional, although frequently used
2. Optional - vasodilator stress-rest perfusion or high dose dobutamine functional imaging to determine the presence of inducible ischemia
3. Optional – aortic imaging and renal MRA to exclude secondary causes of hypertension
4. LGE

#### Left ventricular non-compaction

Goals of imaging LV noncompaction include assessing trabeculations and measuring the wall thickness of compacted and noncompacted segments as well as LV volumes and global function, and assessing for thrombi and scar (LGE)

1. LV structure and function
2. Advanced tissue characterization - optional, although frequently used to exclude other potential etiologies.
3. Optional - vasodilator stress-rest perfusion or high dose dobutamine functional imaging to determine the presence of inducible ischemia
4. LGE

#### Dilated cardiomyopathy

Goals of imaging dilated cardiomyopathy include measuring LV mass, volumes, and global function (by cine imaging), and assessing scar (LGE and T1 mapping).

1. LV structure and function
2. Advanced tissue characterization - optional, although frequently used
3. Optional - vasodilator stress-rest perfusion or high dose dobutamine functional imaging to determine the presence of inducible ischemia
4. LGE

#### Arrhythmogenic ventricular cardiomyopathy (AVC)

Goals of imaging AVC include measuring RV and LV volumes and global and regional function (by cine imaging), and assessing RV and LV scar (LGE).

1. LV structure and function – consider 5–6 mm slice thickness
2. Transaxial or oblique transaxial bSSFP cine images (slice thickness 5–6 mm) covering the RV including RV outflow tract (RVOT). An RV vertical long-axis view aligned with tricuspid inflow is recommended
3. Optional sequences
  - Selected transaxial or oblique transaxial black blood images (double inversion recovery T1-weighted (T1w) FSE)
  - Repeat same geometry with fat suppression
4. LGE. Consider T1 nulling for RV

#### Siderotic cardiomyopathy

Goals of imaging siderotic cardiomyopathy include measuring LV mass, volumes, and global function (by cine imaging), and assessing for iron overload (T2* imaging).

1. LV structure and function
2. Advanced tissue characterization using T2* mapping
3. Optional - vasodilator stress-rest perfusion or high dose dobutamine functional imaging to determine the presence of inducible ischemia
4. Optional - LGE (to be consider if LV or RV ejection fraction is abnormal)

#### Restrictive cardiomyopathy

Goals of imaging restrictive cardiomyopathy include measuring LV mass, volumes, and global function (by cine imaging), and assessing scar and infiltration (LGE and T1 mapping)

1. LV structure and function
2. Advanced tissue characterization - optional, although frequently used
3. LGE
4. Optional (to exclude constrictive physiology) - real time cine imaging, mid-left ventricular short axis, during dynamic breathing manoeuvres for abnormal ventricular interdependence

#### Cardiac sarcoidosis

Goals of imaging sarcoidosis include measuring LV mass, volumes, and global function (by cine imaging), and assessing scar (LGE and T1 mapping), and inflammation/edema (T2w imaging or T2 mapping).

1. LV structure and function
2. Advanced tissue characterization
3. LGE

#### Myocarditis

Goals of imaging myocarditis include measuring LV mass, volumes, and global and regional function (by cine imaging), and assessing for inflammation/edema (T2w imaging or T2 mapping), and increased interstitial space (T1 mapping, LGE).

1. LV structure and function
2. Advanced tissue characterization including techniques listed above
3. Optional - Early Gadolinium Enhancement
4. LGE

#### Cancer-related cardiomyopathies

Goals of imaging cancer-related cardiomyopathy include measuring LV mass and volumes, global function, and maximal wall thickness (by cine imaging), and assessing scar (LGE and T1 mapping). When cardiomyopathy or myocarditis due to chemotherapeutic agents are in consideration, acute/subacute assessment for inflammation/edema (T2w imaging or T2 mapping) may be included.

1. LV structure and function
2. Advanced tissue characterization - optional, although frequently used
3. Optional - vasodilator stress-rest perfusion or high dose dobutamine functional imaging to determine the presence of inducible ischemia
4. LGE

#### Recreational drug-induced cardiomyopathies

Goals of imaging recreational drug-induced cardiomyopathy include measuring LV mass, volumes, and global function (by cine imaging), and assessing scar (LGE and T1 mapping).

1. LV structure and function
2. Advanced tissue characterization - optional, although frequently used
3. Optional - vasodilator stress-rest perfusion or high dose dobutamine functional imaging to determine the presence of inducible ischemia
4. LGE

#### Post-heart transplantation

Goals of imaging post-heart transplantation cardiomyopathy include measuring LV mass, volumes, and global function (by cine imaging), and assessing scar (LGE and T1 mapping) and inflammation/edema (T2w imaging or T2 mapping).

1. LV structure and function
2. Advanced tissue characterization - optional, although frequently used
3. Optional - vasodilator stress-rest perfusion imaging to determine the presence of inducible ischemia
4. LGE

### Vascular disease

#### Peripheral MRA

1. Peripheral vascular coil, or combination of coils, as available
2. Transaxial, low-resolution, vessel scouting with time-of-flight MRA or bSSFP
3. Gadolinium timing
  - Option 1 –A test bolus (transaxial or coronal) at level of distal abdominal aorta. 2 ml injection of GBCA, followed by 20 ml saline. Determine time to peak enhancement following injection using a single-shot bolus tracking sequence
  - Option 2 – Bolus trigger technique to time start of scan
4. Stepping-table, GBCA-enhanced MRA performed in the coronal projection from the mid abdominal aorta to the feet.
  - Two volumetric acquisitions – one pre-contrast (for subtraction) and one during contrast administration
  - GBCA injected in 2 phases to minimize venous contamination followed by saline bolus. See Table 1
  - Slice thickness 1–1.5 mm; acquired spatial resolution in-plane 0.8–1.5 mm
  - Slices – typically 60–100, as needed to accommodate vessels of interest
  - Volumes obtained of abdomen/pelvis and thighs may be coarser spatial resolution (larger vessels), while those of the legs preferably are sub-millimeter spatial resolution. The former acquisitions typically require 15–20 s, while the leg acquisition may take 60–90 s for increased spatial resolution. Elliptical centric k-space acquisition is advantageous for the legs. If available, time-resolved acquisitions are preferred for the legs.
  - Parallel acquisition recommended (multichannel surface coil needed)

Alternative: dual injection protocol

1. Single dose of GBCA: time-resolved MRA of the calf and foot vessels
2. Single dose of GBCA: abdominal and thigh vessels

Alternative: Non-contrast MRA technique

Non-contrast MRA is rapidly evolving and modifications of older methods as well as new techniques are constantly proposed. Some techniques are available for most clinical CMR systems; however as with other sequences, a vendor-specific nomenclature makes general statements difficult. Additionally, many newer techniques are only offered by a limited number of vendors as commercial products.

1. “Fresh Blood Imaging” where two ECG-triggered 3D fast (turbo) spin-echo sequences are performed with the first gated to systole and the second to diastole. Subtraction of the systolic image from the diastolic image set results in an arterial-only image dataset. This is techniques is available for most clinical CMR systems using different vendor-specific acronyms.
  - Slice thickness ~ 2 mm; acquired spatial resolution in-plane 0.6–0.8 mm
  - Slices – typically 40, as needed to accommodate vessels of interest
  - Parallel acquisition recommended (multichannel surface coil needed)
2. 3D bSSFP with an inversion preparation pulse, which provides suppression of background tissue, and with an appropriate TI, allows for the inflow of arterial blood from outside the inversion recovery prepared volume and into the region of interest providing high arterial signal. This is more suited toward smaller volume acquisitions
  - Volume acquired: ~ 340 × 300 × 70; acquired spatial resolution ~ 1.3 × 1.3 × 1.4
  - Parallel acquisition recommended (multichannel surface coil needed)
3. Quiescent Interval slice selective (QISS) MRA is a cardiac gated 2D multi-slice inflow technique, acquired in multiple groups of axial slices with incremental table movement and coverage from pelvis to feet. The sequence uses magnetization preparation pulses to suppress venous flow and stationary tissue and the arterial signal is acquired using a single-shot balanced steady state free precession sequence.
  - Slice thickness 2–3 mm, in plane resolution 1.0–1.2 mm
  - Parallel acquisition routine

#### Thoracic aortic MRA

1. Localizer, 3 orientations
2. Single shot black blood or bSSFP (one breathhold, entire thorax) Transaxial orientation
3. Transaxial T1w FSE or spoiled GRE through aorta (for intramural hematoma, dissection)
4. bSSFP cine imaging in parasagittal plane parallel to and along midline of aorta Option – use 3-point piloting
5. Evaluate aortic valve as per valvular protocol
6. Contrast timing
  - Option 1 -Transaxial/sagittal oblique test bolus in thoracic aorta. 2 ml injection of GBCA, followed by 20 ml saline. Determine time to peak enhancement following injection
  - Option 2 – Bolus triggering technique to time start of scan
  - Option 3 – Rapid multiphase 3D acquisitions without timing
7. 3D GBCA enhanced MRA (0.1–-0.2 mmol/kg
  - Use spatial resolution of at least 1–-1.5 mm
  - Parallel acquisition if available
  - Use ECG gating, if available
  - At least 2 acquisitions after contrast injection
8. Optional - transaxial T1w imaging with fat suppression post-contrast for aortitis
9. Optional – see section 3.2.1 above (Peripheral MRA) for noncontrast MRA techniques

#### Coronary arteries

1. LV structure and function to look for wall motion abnormalities
  - Add repeat horizontal long-axis with high temporal resolution sequence (< < 20 ms per phase) to accurately determine quiescent period of right coronary artery (RCA)
2. Navigator-gated, 3D, free-breathing, MRA sequence:
  - Transaxial slices spanning from level of proximal main pulmonary artery down to the middle of the right atrium (entire cardiac coverage if desired). Slice thickness 1–-1.5 mm; acquired spatial resolution in-plane of 1.0 mm or less. Fat suppression is typically used.
  - Slices – typically 50–-80, as needed to encompass vessels of interest
  - Adjust trigger delay and acquisition window according to observed quiescent coronary period
  - Parallel acquisition preferred
  - Navigator placed over the right hemi-diaphragm
  - Optional – GBCA may increase vessel conspicuity if the contrast agent was administered previously as part of the scan. Due to the relatively long scan time of coronary artery imaging with CMR, a bolus injection is not recommended.
3. Optional –
  - Breathhold techniques if poor image quality or navigators unavailable or of poor quality
  - T2-prepared sequence may be useful to suppress myocardial and venous signal

#### Pulmonary vein evaluation – pre- and post-ablation

1. LV structure and function (optional)
2. Breathheld 3D contrast-enhanced MRA performed in the coronal projection encompassing the pulmonary veins and left atrium (greater anterior coverage if breathholding permits)
  - Optional – oblique plane centering the pulmonary veins can reduce slab thickness and therefore breath hold time but will lead to less coverage of the left atrium
  - Optional - ECG-gating. When patient has irregular rhythm, readout should be synchronized with systole (i.e. no trigger delay)
  - 2–3 volumetric acquisitions – one pre-contrast (for subtraction), one during the first pass of contrast administration, one (optional) after contrast administration
  - Time-resolved multiphase MRA – acquisition and contrast started simultaneously; this can provide isolated pulmonary venous phase image for reconstruction and integration with ablation mapping software
  - GBCA (0.1–0.2 mmol/kg) injected at 2–3 ml/s
  - Slice thickness 1–2 mm; acquired spatial resolution in-plane 1–1.5 mm
  - Slices – typically 60–80, as needed to encompass region of interest
3. Optional – through plane phase contrast flow analysis through each pulmonary vein
4. Optional - LGE of the left atrial wall

### Other

#### Valvular disease

Patients with artificial valves can safely undergo CMR at 1.5 and 3 T. The force exerted by the beating heart is many-fold higher than the force exerted by the magnetic field.

1. General approach
  - Valve morphology assessment with bSSFP cine in the plane of the valve in question. Care must be taken to optimize the level and angle of imaging as described below
  - Note – if planimetry of a stenotic valve is to be attempted, a contiguous or slightly overlapping stack of cine imaging transecting the line of the jet and moving from orifice level to immediately downstream is recommended. Planimetry is most likely to be valid where the cross section of the orifice, or rather of the jet, is clearly delineated.
  - GRE or hybrid GRE-EPI may visualize regurgitant jets with a higher sensitivity (for qualitative purposes only)
  - Velocity encoded imaging to measure velocities and direction quantitatively. Adapt velocity encoding to actual velocity (using lowest velocity without aliasing)
  - Use lowest TE possible for high velocity jet flows
2. Specific approaches by valve
  - Mitral
    i. Regurgitation
      1. LV structure and function
      2. Velocity encoded imaging in a plane perpendicular to the aortic valve, at the sinotubular junction level, at end diastole. Retrospectively-gated acquisition is essential to cover the entire cardiac cycle
    ii. Stenosis
      1. Velocity encoded imaging (though-plane encoding) in a plane parallel to the mitral valve and at the point of peak flow disturbance identified on a long-axis cine image through the mitral valve
      2. Alternatively, velocity encoded imaging (in-plane) along an imaging plane parallel to and in line with the mitral valve jet of flow disturbance
  - Aortic
    i. Regurgitation
      1. LV structure and function
        - Further imaging planned using the planes of the aortic valve and aortic root visualized from LVOT and coronal views.
      2. Velocity encoded imaging in a plane perpendicular to the aortic valve, approximately 5 mm superior to the valve plane at end diastole. Retrospective acquisition is essential to cover the entire cardiac cycle
      3. Velocity encoded imaging in a plane perpendicular to the descending aorta at the level of the main pulmonary artery to examine for diastolic flow reversal
    ii. Stenosis
      1. Velocity encoded imaging (through plane encoding) in a plane parallel to the aortic valve and at the point of peak flow disturbance identified on a long-axis cine image through the aortic valve
      2. Alternatively, velocity encoded imaging (in-plane encoding) along an imaging plane parallel to and in line with the aortic valve jet of flow disturbance
  - Tricuspid
    i. Regurgitation
      1. RV structure and function
      2. Velocity encoded imaging in a plane perpendicular to the pulmonic valve, approximately 5 mm superior to the valve plane, at end diastole. Retrospective acquisition is essential to cover the entire cardiac cycle
    ii. Stenosis
      1. Velocity encoded imaging (through plane encoding) in a plane parallel to the tricuspid valve and at the point of peak flow disturbance identified on a long axis cine image through the tricuspid valve
      2. Alternatively, velocity encoded imaging (in-plane encoding) along an imaging plane parallel to and in line with the tricuspid valve jet of flow disturbance
  - Pulmonic
    i. Regurgitation
      1. RV structure and function
        - Further imaging planned off of pulmonic valve and pulmonic root visualization from RVOT and coronal views
      2. Velocity encoded imaging in a plane perpendicular to the pulmonic valve, approximately 5 mm superior to the valve plane, at end diastole. Retrospective acquisition is essential to cover the entire cardiac cycle
    ii. Stenosis
      1. Velocity encoded imaging (through plane encoding) in a plane parallel to the pulmonic valve and at the point of peak flow disturbance identified on a long-axis cine image through the pulmonic valve
      2. Alternatively, Velocity encoded imaging (in-plane encoding) along an imaging plane parallel to and in line with the pulmonic valve jet of flow disturbance

#### Pericardial disease

1. LV structure and function
2. T1 or T2-weighted FSE images (optional, with or without fat saturation)
  - 2–-3 representative long-axis images and 3–-4 representative short-axis images to measure pericardial thickness (normal ≤3 mm)
  - If pericardial cyst is suspected, refer to masses protocol
3. Optional - iIf regions of thickened pericardium noted – GRE myocardial tagged cine sequences to demonstrate presence or absence of epicardial/pericardial slippage (2–-3 long axis images and 1–-2 short axis images)
4. Real-time imaging during dynamic breathing manoeuvres is valuable for evaluation of ventricular interdependence
  - Mid-ventricular short-axis plane is preferred
  - Cine imaging temporal resolution is preferably below 60 ms
  - Patients are instructed to breathe deeply in and out and the total imaging period should be at least 2 complete respiratory cycles
  - Abnormal septal motion (early diastolic septal flattening or inversion) during onset of inspiration is consistent with a constrictive physiology
5. LGE
  - Acquisition with and without fat saturation is helpful to distinguish pericardial inflammation from epicardial or pericardial fat

#### Cardiac and paracardiac masses, including thrombi

1. LV structure and function
2. T1w FSE - slices through the mass and surrounding structures (number of slices depends on size of the mass)
3. T2w FSE with fat suppression (optional - without fat suppression) - through the mass and surrounding structures as above
4. First pass perfusion module with slices through mass
5. Repeat T1w FSE with fat suppression (early after GBCA)
6. Optional - Repeat selected bSSFP cine images post-contrast
7. LGE
  - Images with the TI set to null thrombus (approximately 500–-550 ms at 1.5 T, 850–-900 ms at 3 T) will help differentiate thrombus from tumor as well as delineate thrombus surrounding or associated with tumor
  - Serial imaging can help distinguish hypoperfused tumor necrotic core from thrombus

## Data Availability

Not applicable.

## Abbreviations

ATP: Adenosine triphosphate
AV: Atrial-ventricular
AVC: Arrhythmogenic ventricular cardiomyopathy
bSSFP: Balanced steady state free precession
CE: Contrast-enhanced
CMR: Cardiovascular magnetic resonance
ECG: Electrocardiogram
ECV: Extracellular volume fraction
EMA: European Medicines Agency
EPI: Echo planar imaging
FDA: Food and Drug Administration
FSE: Fast spin echo
GBCA: Gadolinium based contrast agent
GRAPPA: Generalized autocalibrating partially parallel acquisition
GraSE: Gradient and spin echo
GRE: Gradient echo
HCM: Hypertrophic cardiomyopathy
HR: Heart rate
ICD: Implanted cardiodefibrillator
LGE: Late gadolinium enhancement
LV: Left ventricle/left ventricular
LVOT: Left ventricular outflow tract
MI: Myocardial infarction
MOLLI: Modified Look Locker inversion recovery
MRA: Magnetic resonace angiography
MVO: Microvascular obstruction
NSF: Nephrogenic systemic fibrosis
PSIR: Phase sensitive inversion recovery
QISS: Quiescent interval slice selective
RCA: Right coronary artery
RV: Right ventricle/right ventricular
RVOT: Right ventricular outflow tract
SCMR: Society for Cardiovascular Magnetic Resonance
SENSE: Sensitivity encoding
shMOLLI: Shortened MOLLI
SMASH: Simultaneous acquisition of spatial harmonics
SNR: Signal-to-noise ratio
STIR: Short tau inversion recover
T1w: T1 weighted
T2w: T2 weighted
TE: Echo time
TI: Inversion time
Venc: Velocity encoding
